# New Toxicity Mechanism of Silver Nanoparticles: Promoting Apoptosis and Inhibiting Proliferation

**DOI:** 10.1371/journal.pone.0122535

**Published:** 2015-03-30

**Authors:** Huijing Bao, Xiaoxu Yu, Chen Xu, Xue Li, Zhaoyang Li, Dianjun Wei, Yunde Liu

**Affiliations:** 1 School of Laboratory Science, Tianjin Medical University, Tianjin, China; 2 The 4^th^ Central Hospital, Tianjin, China, China; 3 School of Materials Science and Engineering, Tianjin University, Tianjin, China; 4 The Department of Laboratory Science, The Second Hospital of Tianjin Medical University, Tianjin, China; National Cheng Kung University, TAIWAN

## Abstract

Silver nanoparticles are increasingly recognized for their utility in biological applications, especially antibacterial effects. Herein, we confirmed the antibacterial effect of silver nanoparticles on *Escherichia coli* using the conventional optical density (OD) and colony-forming units (CFU) method and used flow cytometry (FC), TEM and BrdU ELISA to investigate the mechanisms of this effect. From the results, we conclude that AgNPs can simultaneously induce apoptosis and inhibit new DNA synthesis in the cells in a positive concentration-dependent manner. This study presents the first induction of apoptosis in these bacteria by AgNPs in this field. Our findings may provide a new strategy for the use of silver nanoparticles in antibacterial applications.

## Introduction

Silver is used as a strong inhibitor for a broad spectrum of antimicrobial activities, such as those of bacteria, fungi, and viruses. Compared with other metals, silver shows higher toxicity to microorganisms while exhibiting lower toxicity to mammalian cells[1]. It has been conformed that Ag^+^ ions, a prototypical antimicrobial silver species in the form of a silver nitrate solution, are active against a wide range of bacteria and fungi[2]. Nanometer-sized silver particles (AgNPs) have long been known to have an antibacterial effect. AgNPs are generally smaller than 100 nm, containing 20–15,000 silver atoms, and exhibit unusual physical, chemical and biological properties[3]. Due to their strong antibacterial activities, the use of AgNPs and their composites has been suggested for preventing bacterial infection in surgery[4], in the coatings of medical devices[5,6] or even as a water disinfectant or room spray[3]. However, the mechanisms of this antibacterial effect are unclear. The most widely known mechanism of AgNPs is the inhibition of the enzymatic function of some proteins by interaction with the thiol groups of L-cysteine [7–9]. Promoting the permeability of the bacterial membrane [1] and disrupting the membrane integrity [10] are also thought to be responsible for the antibacterial effect. Moreover, it has been discovered that silver can bind to the DNA, increasing the decomposability of genome DNA[11–13] or inactivating the respiratory chain, inducing the formation of hydroxyl radicals[9].

In previous studies, the antibacterial mechanism of AgNPs has only been partially elucidated. Programmed cell death (PCD), which induces apoptosis, is an essential mechanism in eukaryotic organisms[14] and also can been found in prokaryotes cells, such as *E*.*coli* cells [15]. In our work, a new mechanism of the antibacterial activity of AgNPs was identified. For the first time, we demonstrate the antibacterial mechanism of AgNPs in terms of inducing bacterial apoptosis.

## Materials and Methods

### Reagents and antibodies

AgNP solution 100AGS-WMB1000C (diameter: 5~10 nm, concentration: 1000 ppm) was purchased from Shanghai Huzheng Nanotechnology Co., Ltd. The propidium iodide (PI) reagent (50 μg/ml) was purchased from BD Co. Bovine serum albumin (BSA) was produced from Sigma Co. The FITC-conjugated annexin V and PI kit was obtained from Dojindo Molecular Technologies, Inc. The cell proliferation kit was purchased from Roche Co.

All other chemicals were supplied by Aldrich and used as received. The *E*. *coli* strain (ATCC 25922) was purchased from American Type Culture Collection (ATCC) and conserved in our laboratory. The FACS buffer was prepared with 0.5% BSA, 2 mM EDTA and 500 ml PBS. Luria-Bertani (LB) liquid medium and solid medium were prepared in our laboratory.

### Nanoparticle characterization by TEM

The morphology of the AgNPs was characterized by an analytical transmission electron microscope (TEM). Aliquots of the AgNP solutions (5 and 10 μg/ml) were dropped onto the carbon-coated copper (Cu) grid and then air-dried before TEM observation. The chemical analysis of the AgNP solutions was performed using the energy dispersive x-ray spectroscopy (EDX) module attached to the TEM (JEOL JEM-2100).

### Antibacterial effect of AgNPs measured by OD_600_ and CFU

The *E*. *coli* cells were cultured in 5 ml of LB medium at 37°C overnight. After incubation, the cells were diluted (1:100) in 300 ml of LB medium and incubated with 5 or 10 μg/ml AgNPs at 37°C and 220 rpm for 24 h. The bacterial concentrations were determined by both measuring the optical density (OD) and counting colony-forming units (CFU). The absorbance was determined at 600 nm by spectrophotometry (Beijing Purkinje General Instrument Co., Ltd., China). Each experiment was performed twice, and the growth curves were plotted by Prism 5 software (http://www.graphpad.com/).

### Flow cytometry analysis of dead bacteria

The *E*. *coli* cells were cultured overnight and then incubated with 300 ml of LB (under 1:100 dilution) containing 5 or 10 μg/ml AgNPs for 1, 2 and 3 h. At each time point, the cells were spun down at 10000g for 10 min and resuspended in 500 μl of FACS buffer. Next, 2 μl PI was added to each sample, and the samples were incubated for 10 min at room temperature. The red fluorescence was measured by a FACSVerse (BD) instrument. Experiments were performed in triplicate, and the data were analyzed using *FlowJo* software (http://www.flowjo.com/). Cells with 70% isopropanol treated for 1 h were used as the positive control.

### TEM analysis of the morphology of AgNP-treated bacteria

The morphology of the bacteria treated with silver nanoparticles was observed using TEM. *E*. *coli* cells were exposed to AgNPs (5 or 10 μg/ml) and collected at 3 h. A drop of the bacteria sample solution was placed on a carbon-coated copper grid and dried in air at room temperature before TEM observation.

### Flow cytometry analysis of bacteria apoptosis

The *E*. *coli* cells were cultured overnight, diluted 100 times and cultured in 5 ml of LB medium containing 5 or 10 μg/ml AgNPs for 1, 2 and 3 h. Bacteria cells were collected at each time point and washed once with 1 ml of cold, filtered 1×PBS. Next, the cells were resuspended in 100 μl of 1×annexin V-binding buffer (kit component) and incubated with 5 μl of FITC-conjugated annexin V and 2 μl of PI for 15 min at room temperature. To detect PS exposure by flow cytometry, samples were diluted by adding 400 μl 1×annexin V-binding buffer and 500 μl 1×PBS and then placed on ice. Data were collected by a FACSVerse instrument. Experiments were performed in triplicate, and the data were analyzed by *Flowjo* software. Isopropanol (70%)-treated cells were used as the PI positive control, and ampicillin (5 μg/ml)-treated cells were used as the annexin V positive control.

### BrdU ELISA study of bacterial proliferation

Bacterial proliferation was determined by measuring BrdU incorporation using a BrdU ELISA kit according to the manufacturer’s instructions. Briefly, *E*. *coli* cells were co-cultured with AgNPs (5 and 10 μg/ml) and BrdU (10 μM) for 1, 2 and 3 h. At each time point, 200 μl of cells was collected, spun down at 8000 rpm for 10 min, resuspended in 100 μl of peroxidase-conjugated anti-BrdU antibody and incubated for 30 min. The absorbance values were measured at 370 nm using a microplate reader (Biotek, USA). The experiments were performed in triplicate, and the growth curves were plotted by Prism 5 software.

### Statistical analysis

One-way ANOVA and Tukey’s multiple comparison test was used to determine whether the nanoparticles had any significant effect on the cells. Statistically significant differences were identified when *P*<0.05.

## Results

### Nanoparticle characterization

**Fig 1:**
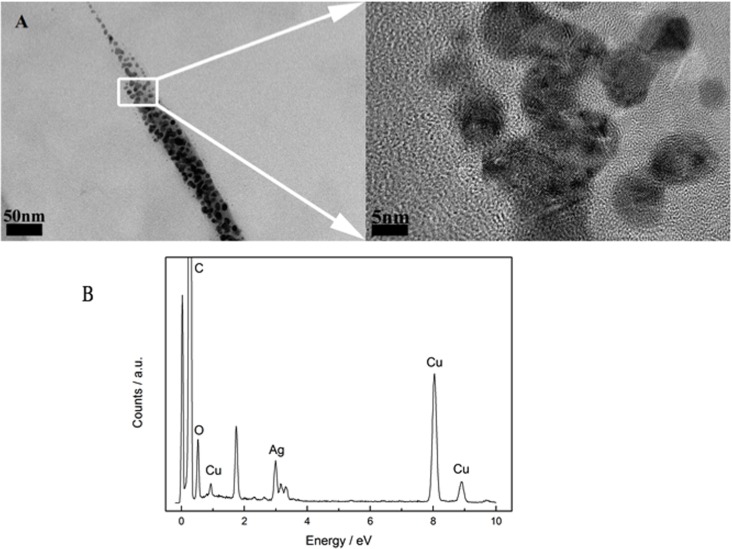
The analysis of AgNPs Characterizations by TEM. A. The AgNPs show the regular nanoparticle shape with the diameter ranging from 5 to 10nm. B. The elemental silver was conformed by EDX spectrum during the TEM experiment.

Transmission electron microscopy (TEM) observation revealed the morphology and size of the AgNPs in solution, which were spheres 5~10 nm in diameter (Fig. 1A). To confirm the content of the AgNP solution, chemical analysis was performed using the EDX spectra. Only carbon, copper and silver were found in the EDX results, and the carbon and copper are from the carbon-coated copper (Cu) grid. So the results confirmed the solutions used in the subsequent experiments contained silver nanoparticles (Fig. 1B).

### Bacteria growth curve

**Fig 2:**
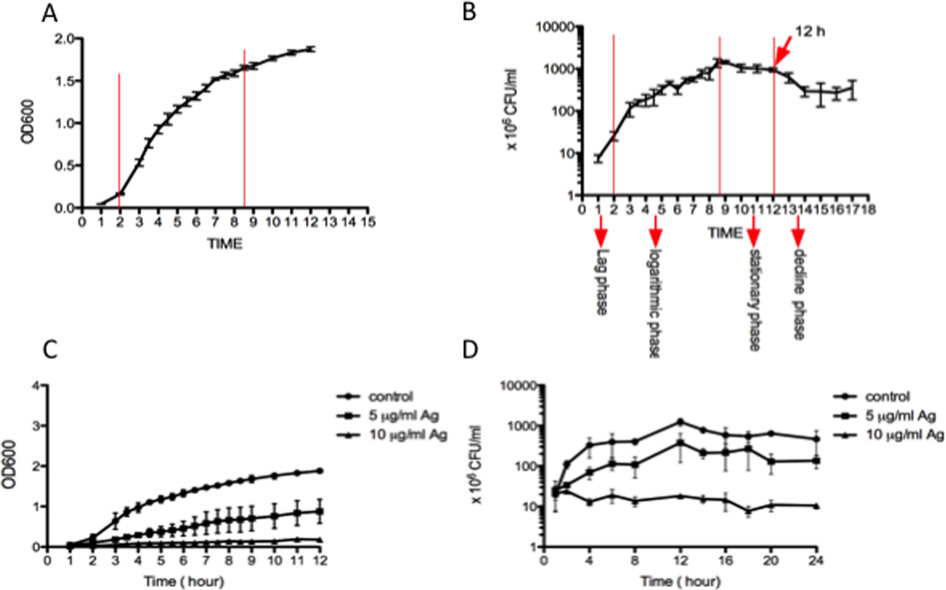
Determination of *E*. *Coli*. growth by taking the absorbance at 600 nm and counting CFU. A. The normal growth curve by analyzing the absorbance at 600 nm. B. The normal growth curve by counting the CFU. C. The assessment of toxicity of NP-Ag in *E*. *Coli*. growth by plotting the curve of absorbance. D. The toxicity of NP-Ag in *E*. *Coli*. growth by CFU counting.

In most experiments, the antibacterial toxicity of the nanoparticles is measured by the optical density at 600 nm. However, no evidence was available for the relationship between the optical density and actual growth (CFU) for the bacterial throughout the growth period. In our experiments, both the optical density and CFU were used to evaluate the non-stimulated bacterial growth to investigate the relationship between these two methods. The standard growth curve of the non-stimulated bacteria, including the lag, log, stationary and decline phases, was observed using the CFU method. The log phase appeared after 2 h and lasted for 7 h, and the stationary phase was observed over the next 3 h. After 12 h, the curve exhibited a decrease in bacterial growth, corresponding to the decline phase (Fig. 2B). The optical density increased with the growth time, and no standard growth curve was observed using this method. This increasing trend was observed even after entering the decline phase, as the bacteria began to die (Fig. 2A). These results showed that optical density measurements could only be used to calculate bacterial growth before the decline phase, which began at 12 h in our experiments.

In our experiments, the toxicity of AgNPs was also analyzed by optical density and CFU. The OD_600_ and CFU results demonstrated that after different incubation times with different AgNP concentrations, the cells showed higher toxicity than the control sample (Fig. 1C and 1D). The control samples, which were not treated with AgNPs, exhibited a standard CFU growth curve and a continuous increase of the OD_600_ before the decline phase. At each time point, the AgNP-treated cells presented a lower optical density and CFU than the control sample. This decreasing trend for the stimulated sample is positively correlated with the AgNP concentration. All of these results indicated that the AgNPs could decrease the number of *E*. *coli* cells, the mechanism of which will be determined by assaying.

### Analysis of dead bacteria

**Fig 3:**
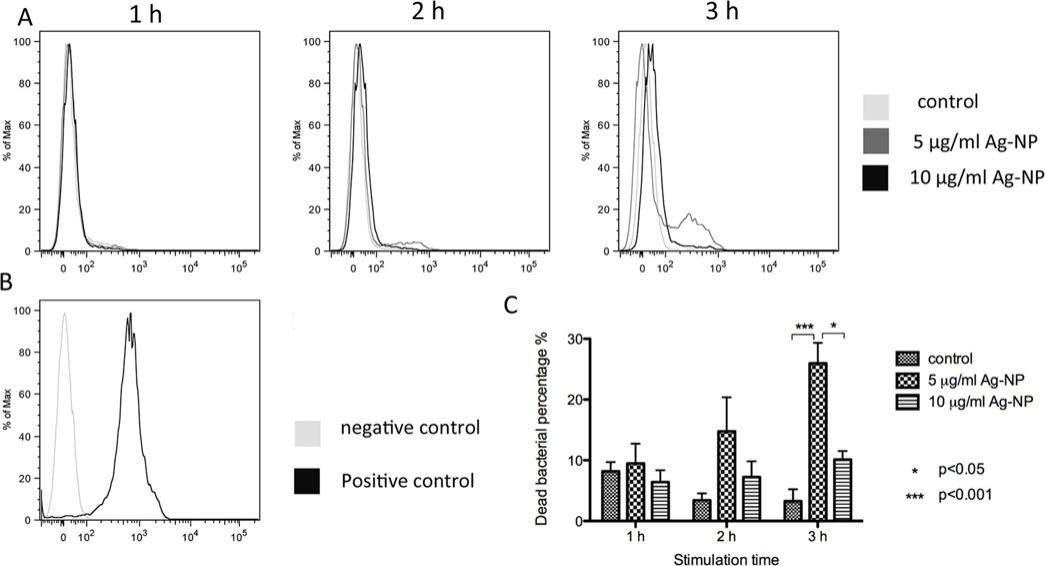
The analysis of dead ratio in bacterial cells by flow cytoometric. A. Flow cytometric analysis for dead bacterial percentage using PI staining. B. Flow cytometric analysis control: negative control using bacteria shaken 1h without any treatment and positive control using bacteria co-cultured with 70% isopropanol. C. statistic analysis by one way ANOVA test and Tukey’s multiple comparison test. (n = 3).

The number of *E*. *coli* cells decreased after incubation with the AgNP solution, indicating more dead cells should be found in the stimulated group. We used flow cytometry to analyze the dead cell percentage in each group using PI dye. The positive peak height signal is enhanced for the AgNP (5 or 10 μg/ml)-treated bacteria relative to the control group, suggesting that more dying cells were found in the stimulated group. However, the signal of the 5 μg/ml-stimulated group is much higher than that of 10 μg/ml group, indicating that the lower-concentration group yielded a higher proportion of dead cells (Fig. 3A and 3B). Statistical analysis by one-way ANOVA followed by Tukey’s test revealed that the 5 μg/ml AgNPs had statistically significantly higher toxicity to the bacterial cells after 3 h of stimulation (Fig. 3C).

### Effect of stimulation on bacterial morphology

**Fig 4:**
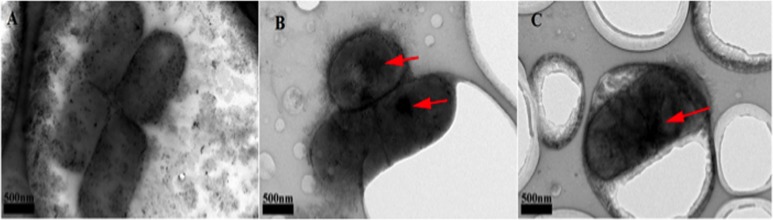
The observation of the micrograph of *E*. *coli* cells by TEM. A. Control group with no AgNPs stimulated. The typical morphology of *E*. *coli* was observed with the integrity cell membrane. B. *E*. *coli* stimulated with 5 μg/ml AgNPs group. The integrity cell membrane with no significant obvious damage can be found. Besides that, several electron dense granules also can be observed (the red arrow). C. *E*. *coli* stimulated with 10 μg/ml AgNPs group. There are also electron dense granules in *E*. *coli* (the red arrow), and the cell membrane also show the integrity.

The morphology of the stimulated bacteria was observed by TEM. The control samples exhibited the typical morphology of *E*. *coli* cells and an even distribution of the DNA inside the cell (Fig. 4A). Electron-dense particles were found after stimulation with AgNPs (5 or 10 μg/ml) for 3 h; however, the bacterial cell wall and bacillus morphology was typical and integrated (Fig. 4B and 4C). These results suggest that the toxicity of the AgNPs is the result of action inside, not on, the bacteria.

### Apoptosis of the bacteria

**Fig 5:**
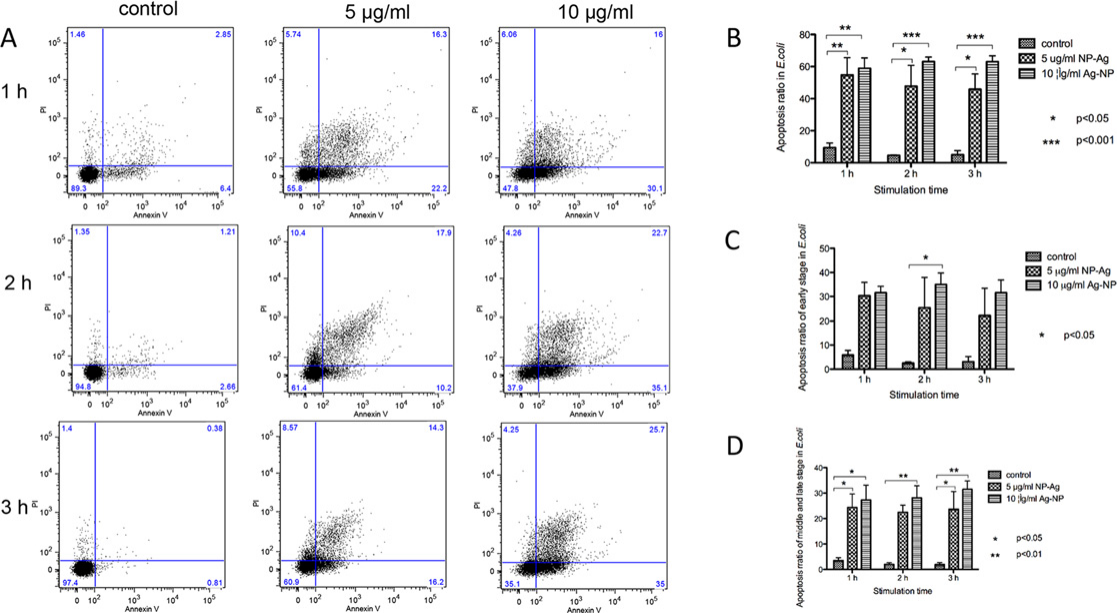
The analysis of the apoptosis in the cells. A. Flow cytometric analysis for apoptosis bacterial percentage using Annexin V-PI staining (one experiment results). B, C and D. statistic analysis for apoptosis bacterial percentage by One-way ANOVA test, followed Tukey’s multiple comparison test (n = 3). B shows the total percentage of apoptosis bacteria. C shows the percentage of apoptosis bacteria in early stage, including the bacteria in the PI-negative Annexin V-positive quadrant. D shows the percentage of apoptosis bacteria in middle and late stage, including the bacteria in PI-positive Annexin V-positive quadrant.

The apoptosis of the bacteria was analyzed by an apoptosis kit, including PI and FITC-labeled annexin V dye. Phosphatidylserine (PS) exposed outside the cell during the apoptosis stage can be bound with annexin V dye with high specificity. The results showed that the apoptosis number in the cells increased after stimulation with the AgNP solutions and that apoptosis also increased with the concentration of the AgNP solution (Fig. 5A). The statistical analysis by one-way ANOVA followed by Tukey’s test evidenced the statistical significance of these results. The P values of the apoptosis rate between the control group and the 5 or 10 μg/ml groups were significant (Fig. 5B), and the apoptosis rate increased with the AgNP concentration. The apoptosis process can be divided into early, middle and late phases. According to the FC results, the apoptosis rate in all three stages was higher for the stimulated group than the control group, and this rate increased with the AgNP concentration (Fig. 5C and 5D).

### Bacteria proliferation

**Fig 6:**
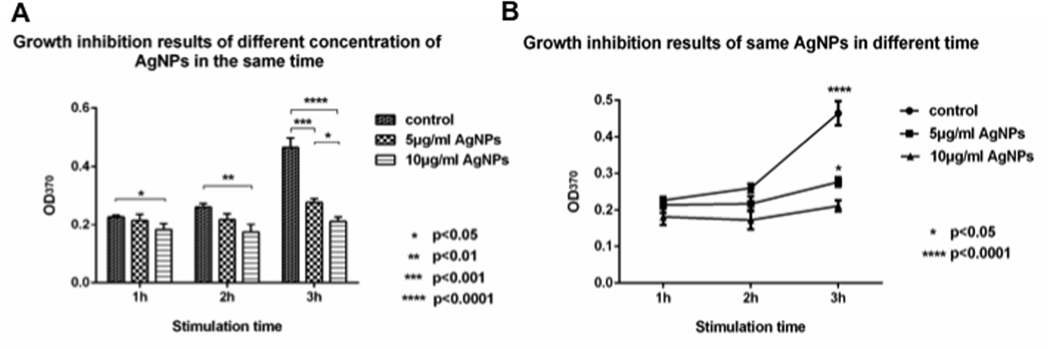
The new DNA systhesis analysis by ELISA. A. growth inhibition results of different concentration of AgNPs at the same time. B. Growth inhibition for the same concentration of AgNPs at different times. All data was analyzed by One-way ANOVA test and Tukey’s multiple comparison test following (n = 3).

To monitor the tendency of bacterial proliferation via the synthesis of new DNA, a BrdU proliferation kit was used. Newborn DNA can be combined with BrdU dye and tested at 370 nm using an absorbance reader. The results showed that the absorbance of the AgNP (5 or 10 μg/ml)-treated bacteria was lower than that of the control group and that the reduction in new DNA synthesis was related to the AgNP concentration (Fig. 6A). The higher AgNP concentration yielded a lower new DNA synthesis rate (Fig. 6B). These data were analyzed by one-way ANOVA, revealing a statistically significant difference between the stimulated and control groups.

## Discussion

Pathogenic bacteria show great resistance to various anti-infective agents. Furthermore, the alarming growth of antibiotic-resistant superbugs, such as vancomycin-resistant *Staphylococci*, has become a major global health hazard[16]. The increasing emergence of antibiotic resistance among pathogenic bacteria has led to the search for alternatives to overcome this alarming problem[17]. With the rapid development of nanotechnology, a great variety of nanoparticles are entering application in our practical lives, most of which have been proven to be toxic to bacteria[18]. However, the antibacterial mechanism of AgNPs has not been completely elucidated. Therefore, studying the mechanism of this antibacterial effect is very important.

In our works, the growth curves of *E*. *coli* exposed to AgNPs, as shown in Fig. 2, indicate that AgNPs can influence the number of *E*. *coli* by decreasing the growth rate. This decreasing trend is related to the concentration of the AgNPs and can be attributed to the increase in the percentage of dead bacterial cells after co-culture with the AgNPs. To understand this phenomenon, PI dye was used to analyze the percentage of dead bacterial cells after treatment with AgNPs. Compared with the control group, the PI positive percentages of the bacterial cells in the stimulated groups (5 μg/ml and 10 μg/ml AgNPs) are much higher and reach statistical significance after 2 h. However, this increasing PI positive percentage is not related to the concentration of the AgNPs, and the highest positive ratio of the bacterial cells was observed in the 5 μg/ml group, which is the opposite of the result of the growth curve study. The PI dye works by entering cells whose membrane is structurally damaged and then anchoring to nucleic acid. This membrane damage could be due to cell necrosis or apoptosis, especially the middle and late stages of apoptosis. Therefore, one reason for the high PI positive ratio in the 5 μg/ml groups may be the greater damage of the bacteria membrane. As the CFU is higher in the 5 μg/ml group than the 10 μg/ml group, the PI positive ratio may be higher in the 5 μg/ml group due to membrane damage unrelated to necrosis. To verify our hypothesis, the bacterial morphology was observed by TEM. After stimulation with AgNPs (5 μg/ml or 10 μg/ml), although several electron-dense granules were observed in the center of the bacterial cells, most of the cells membranes are smooth and intact. Furthermore, there is no significant evidence supporting that the membrane damage is more severe in the 5 μg/ml group. These results indicate that this membrane damage could be from cell apoptosis.

Recently, bacteria apoptosis has received increasing attention. In 2012, Daniel found that antibiotic-induced bacterial cell death exhibited the physiological and biochemical hallmarks of apoptosis[19]. At the same time, several researchers also reported observations of apoptosis in bacteria[20,21]. The exposure of PS on the outer leaflet of the plasma membrane of cells is a defining biochemical marker of apoptosis[22]. An annexin V/PI double dye kit for apoptosis was used in our experiments to analyze the apoptosis during the AgNP stimulation. According to the results from Fig. 5, AgNPs can induce apoptosis in the *E*. *coli* cells, and this activity increases with increasing AgNP concentration. The analysis of each stage of cell apoptosis yields the same conclusion. Higher AgNP concentrations induce greater apoptosis. This phenomenon can also be observed from the early stage to the late stage of apoptosis, indicating that the membrane damage due to apoptosis is not greater in the 5 μg/ml group than in the 10 μg/ml group.

The above results demonstrate that the AgNPs can induce apoptosis in the *E*. *coli* and that this trend is positively related to the AgNP concentration. However, the higher PI positive ratio for a lower apoptosis ratio in the 5 μg/ml group still cannot be explained. According to the PI dye working mechanism, in addition to cell membrane damage, the staining results also can be affected by the quantity of the DNA inside the cells. Moreover, the apoptosis, which has already been proven to occur in *E*. *coli* cells after stimulation by AgNPs, can induce filamentation, which is a marker of cell division arrest in *E*. *coli*[19,23]. Thus, we suspect that the AgNPs can also inhibit bacteria proliferation by inhibiting DNA synthesis.

Newborn DNA synthesis was analysis by the BrdU ELISA kit. According to the results from Fig. 6, the new DNA synthesis was inhibited by the AgNPs. The extent of this inhibition was also positively related to the nanoparticle concentration. This indicates that the number of newborn DNA in the 5 μg/ml group is higher than that in the 10 μg/ml group. In other words, the DNA content of the 5 μg/ml group is higher than that of the 10 μg/ml group. For the same extent of cell membrane damage, the higher the DNA content, the higher the PI positive ratio. Thus, the higher newborn DNA synthesis in the 5 μg/ml group the 10 μg/ml group is the cause of the higher PI positive ratio for a lower apoptosis ratio.

In conclusion, our results shed light on the toxicity of AgNPs to bacteria, which occurs via the induction of bacteria apoptosis and inhibition of newborn DNA synthesis. The extents of these effects are positively related with the AgNP concentration. These results can be used to develop new antibacterial strategies for AgNPs. However, the specific target of the AgNPs in the inhibition of the newborn DNA synthesis or the induction of apoptosis has not been identified. Thus, the molecular mechanism needs to be further investigated.
